# Weight Regain After GLP-1-Based Therapy Discontinuation: Failure, Physiology, or Follow-Up Gap

**DOI:** 10.7759/cureus.104259

**Published:** 2026-02-25

**Authors:** Andres F Quimbayo-Cifuentes

**Affiliations:** 1 Clinical Epidemiology, Universidad Instituto Colombiano de Estudios Superiores de Incolda (ICESI), Santiago de Cali, COL; 2 Geriatrics, Sociedad Integral de Especialistas en Salud, Armenia, COL; 3 Internal Medicine, Centros Medicos Colsanitas Sociedad por Acciones Simplificada (SAS), Armenia, COL

**Keywords:** glp-1 receptor agonists, obesity, obesity treatment, overweight and obesity, rebound effect

## Abstract

The introduction of glucagon-like peptide-1 receptor agonists (GLP-1 RAs) and dual incretin agonists targeting both the GIP and GLP-1 receptors (GIP/GLP-1 dual agonists) has reshaped obesity management, approaching degrees of weight loss previously achievable largely through metabolic surgery. However, randomized withdrawal trials, including Semaglutide Treatment Effect in People with Obesity (STEP) 4 and SURMOUNT 4, show that discontinuation of GLP-1-based therapy is consistently followed by rapid weight regain (typically observed within one year of withdrawal) and a decline in cardiometabolic benefits. Rather than indicating therapeutic failure, this pattern is best understood as disease recurrence, reinforcing obesity as a chronic, relapsing condition. After treatment is discontinued, homeostatic weight-defense mechanisms re-emerge, favoring a return toward the pre-treatment set point. This editorial examines the biology underpinning post-discontinuation weight regain and highlights a clinically underappreciated consequence: sarcopenic obesity, driven by preferential fat mass recovery relative to lean mass. It also discusses mitigation strategies, including resistance training and structured tapering approaches, and argues for long-term, maintenance-oriented care as an ethical imperative in chronic obesity management.

## Editorial

Recent advances in anti-obesity pharmacotherapy have expanded therapeutic possibilities while also sharpening questions about what follows when treatment stops [1,2]. Although the efficacy of semaglutide and tirzepatide is well established, the term “rebound effect” is still commonly used to describe post-discontinuation weight regain [2]. The term is scientifically imprecise and subtly stigmatizing, implying individual failure rather than predictable disease biology; it is more accurately described as disease recurrence, consistent with obesity as a chronic, relapsing condition [3]. As with hypertension, asthma, or the recurrence of depressive symptoms after discontinuing selective serotonin reuptake inhibitors (SSRIs), in which control predictably deteriorates when therapy is stopped, obesity's underlying physiology reasserts itself when GLP-1-based treatment is discontinued. Randomized withdrawal data show a consistent pattern: discontinuation is followed by clinically meaningful weight gain and attenuation of cardiometabolic benefit, underscoring that these agents function as long-term disease-modifying therapies rather than curative interventions [1,4,5].

The biological drivers of this recurrence are complex yet largely predictable. GLP-1-based therapies reduce appetite and modulate reward-related eating; however, discontinuation does not restore a neutral physiological baseline [6]. Instead, it unmasks counter-regulatory responses, such as increased orexigenic signaling (including ghrelin) and metabolic adaptation, that favor replenishment of energy stores. This post-withdrawal physiology is often asymmetric: orexigenic signals rise and satiety signals fall, while energy expenditure remains suppressed relative to body size, together reinforcing the defended set point [6,7]. Randomized withdrawal trials make this pattern clear. In the Semaglutide Treatment Effect in People with Obesity (STEP) 4 trial, participants who transitioned to placebo after a 20-week run-in exhibited early weight gain, whereas those who continued active treatment maintained benefit [5]. Similarly, in the SURMOUNT 4 trial, discontinuation of tirzepatide was associated with substantial weight regain, with participants regaining approximately 14% of their body weight on average within the 52-week follow-up period, and attenuation of the initial metabolic improvements [4]. The propensity for recurrence may be greatest among high responders, in whom the physiological distance between nadir weight and the pre-treatment set point is largest [2].

Even when weight regain is expected as part of disease recurrence, body weight alone does not capture the full clinical consequence of discontinuation. A clinically important concern is the direction of body-composition change. During weight loss, a meaningful fraction of the reduction represents lean body mass (LBM) [8]. The challenge is the asymmetry of recovery: once therapy is discontinued, fat mass is restored more rapidly and efficiently than skeletal muscle, particularly in the absence of a sustained anabolic stimulus [9]. This mismatch presents a theoretical risk for a post-treatment phenotype with higher adiposity and lower LBM than baseline, consistent with sarcopenic obesity [10]. While further long-term body composition studies are needed to confirm if LBM recovery consistently lags behind fat mass across all populations, the implications are especially important in older adults, where diminished reserve amplifies insulin resistance and increases vulnerability to frailty and functional decline [11].

Consequently, obesity management must move beyond episodic prescribing toward a longitudinal, maintenance-oriented model. Abrupt discontinuation should be avoided whenever feasible. Emerging evidence suggests that structured tapering or extended-interval dosing (e.g., every 10-14 days in selected patients) may offer pragmatic maintenance options when access, cost, or tolerability threaten continuity, although robust long-term efficacy data remain limited and further validation in controlled trials is needed [12]. In parallel, clinicians should establish a clear therapeutic contract that prioritizes preservation of lean body mass through adequate protein intake and progressive resistance training [8]. These measures are not optional adjuncts; they are core components of a strategy to sustain metabolic benefit and protect functional capacity over the long term. Given that cost and access are leading drivers of discontinuation in real-world settings, health systems share responsibility for ensuring coverage and continuity of maintenance therapy when clinically indicated - an obligation that aligns directly with the ethical imperative to treat obesity as a chronic disease [13].

Ultimately, weight regain after treatment cessation reflects a core feature of obesity biology rather than an exception. Health systems and clinicians should move away from stigmatizing recurrence and instead design care around long-term disease management. If cardiometabolic markers reliably drift back toward baseline after therapy is stopped, then short-term, induction-only approaches are misaligned with the condition being treated. In many cases, the failure lies neither in the medication nor the patient, but in a therapeutic framework that lacks durable maintenance and transition plans for a disease that requires lifelong management.
